# Ab Initio Neural Network Potential Energy Surface and Quantum Dynamics Calculations on Na(^2^S) + H_2_ → NaH + H Reaction

**DOI:** 10.3390/molecules29204871

**Published:** 2024-10-14

**Authors:** Siwen Liu, Huiying Cheng, Furong Cao, Jingchang Sun, Zijiang Yang

**Affiliations:** School of Physics and Electronic Technology, Liaoning Normal University, Dalian 116029, China

**Keywords:** potential energy surface, ab initio calculations, neural network, quantum dynamics, time-dependent wave method

## Abstract

The collisions between Na atoms and H_2_ molecules are of great significance in the field of chemical reaction dynamics, but the corresponding dynamics results of ground-state reactions have not been reported experimentally or theoretically. Herein, a global and high-precision potential energy surface (PES) of NaH_2_ (1^2^*A*′) is constructed by the neural network model based on 21,873 high-level ab initio points. On the newly constructed PES, the quantum dynamics calculations on the Na(^2^S) + H_2_(*v*_0_ = 0, *j*_0_ = 0) → NaH + H reaction are carried out using the time-dependent wave packet method to study the microscopic reaction mechanism at the state-to-state level. The calculated results show that the low-vibrational products are mainly formed by the dissociation of the triatomic complex; whereas, the direct reaction process dominates the generation of the products with high-vibrational states. The reaction generally follows the direct H-abstraction process, and there is also the short-lived complex-forming mechanism that occurs when the collision energy exceeds the reaction threshold slightly. The PES could be used to further study the stereodynamics effects of isotope substitution and rovibrational excitations on the title reaction, and the presented dynamics data would provide an important reference on the corresponding experimental research at a higher level.

## 1. Introduction

The interactions of alkali atoms with hydrogen molecules and the corresponding isotopic variants, including both reactive collisions and nonreactive quenching, have become important objects in studying chemical reaction dynamics due to the unique quasi-one-electron nature of alkali atoms, which make accurate theoretical calculations feasible and experimental operability. Among these alkali elements, the reactions between electronically excited Na or Li and H_2_ follow surface-hopping mechanisms [1,2,3,4], yielding the products using a vertical insertion approach. In contrast, the electron harpoon model dominates the high-lying K, Rb, and Cs reaction processes with H_2_ [5,6,7,8]; namely, most of the excess energy is transferred into translational states.

The Na + H_2_ collision, as a simple prototype of such systems, has been widely studied in the last few decades, and various experimental results are available. Bililign and Kleiber used the laser-pump-probe technique to determine the nascent rotational quantum state distributions of NaH and NaD molecules generated by the Na(4^2^P) + H_2_/D_2_/HD reactions [9]. The experimental results showed a bimodal rotational distribution with the major and minor components peaking at high and low rotational states, respectively, and the isotope substitution had little influence on the rotational distribution. This research implies that the reaction occurs predominantly on the attractive 2*B*_2_ surface near *C*_2*v*_ symmetry. This study also measured the far-wing absorption profiles of the NaH_2_ intermediate, resulting in the nonreactive formation of Na and four rotational states of the reactive product NaH (*j* = 3, 4, 11, and 13) [1]. Combined with the experimental measurements and theoretical analysis, it can be concluded that the effect of the Na reagent electronic orbital alignment on the final product rotational distribution—namely, absorption to the repulsive surface and attractive surface—lead preferentially to low and high rotational states, respectively. Motzkus et al. applied three different nonlinear optical techniques, CARS, resonance-enhanced CARS, and degenerate four-wave mixing to compare the quenching and reactive behavior of the Na(4p) + H_2_ and Na(3p) + H_2_ processes [10]. The results presented that the formation of NaH products proceeds indirectly in the Na(3p) + H_2_ collisions as opposed to the direct reaction of Na(4p) + H_2_, and the corresponding maximum of the vibrational population is at *v*′ = 0 and *v*′ = 1 of the NaH molecule, respectively. In 2008, Chang et al. obtained the nascent rotational and vibrational state distributions of NaH molecules formed by the reactions of Na(4^2^S, 3^2^D and 6^2^S) with H_2_ based on a pump-probe technique [11]. Similar to the case of the Na(4^2^P) + H_2_ reaction, the distributions of rotational state for Na(4^2^S, 3^2^D) reactions displayed a bimodal feature with a main component peaking at *j*′ = 20–22, while the Na(6^2^S) reaction showed a different rotational distribution with a much lower temperature. The collisions of the 4^2^S and 3^2^D states are initiated along the entrance surface in a near *C*_2*v*_ geometry and then transition to the reactive 1*A*′ surface. In contrast, the reaction of the 6^2^S state features a reduced ionization energy and follows a harpoon-type mechanism through a near-collinear configuration. 

In the theoretical aspect, numerous ab initio results and dynamics calculations on the NaH_2_ system have been also reported. Botschwina et al. calculated the first potential energy surfaces (PESs) for the four lowest states that were used to study the nonreactive collision of the Na(3p) + H_2_ process using the RHF-SCF and PNO-CEPA methods [12], and the dependence of total quenching cross-sections on the initial collision energy was obtained employing the “absorbing sphere” model. Donald et al. represented the three lowest PESs and the nonadiabatic couplings for the Na(3p) + H_2_ quenching collisions based on the diatomics-in-molecules model [13], and the PESs were optimized by a new parametrization for the potential energy curve of the NaH diatomic molecule [14]. In 1985, Yarkony reported the first ab initio treatment of the nonadiabatic coupling strengths for the Na(^2^P) + H_2_ → Na(^2^S) + H_2_ quenching process at the state-averaged MCSCF/CI level [15], which included the conical intersections of the 1^2^*A*′ and 2^2^*A*′ states at *C*_2*v*_ geometry, and the matrix elements of nonadiabatic couplings were determined based on an analytic gradient algorithm. Halvick and Truhlar presented a diabatic coupled PES for the Na(3p) + H_2_ → Na(3s) + H_2_ or NaH + H processes based on the modified eight-state diatomics-in-molecules representation [16]. In 1999, they constructed a new 2 × 2 diabatic potential energy matrix based on the ab initio calculations at the MP2 level with the aug-cc-pCTVZ and cc-pCVDZ basis sets for H and Na atoms, respectively, which covered a small number of points near the three-body interaction region [17]. Due to the limitations of early calculation conditions and fitting methods, the previously reported PESs may not be used to accurately study the reaction dynamics characteristics. In 2018, we structured a set of globally accurate two-state coupling diabatic NaH_2_ PESs using the artificial neural network (NN) model based on a mass of data obtained by a molecular-property-based diabatic transformation of high-level ab initio point, and the quantum dynamics of the Na(3p) + H_2_ → NaH + H reaction were carried out to study the reaction mechanism in detail [18]. On the new diabatic PESs, several collision dynamics calculations on the NaH_2_ system have been performed in recent years [19,20,21,22,23,24]. Buren et al. studied the intramolecular isotope effects of the Na(3p) + HD reaction using the time-dependent wave packet (TDWP) method [21], and a new insertion–abstraction mechanism was proposed based on the calculated results of two reaction channels. Zhang et al. implemented the TDWP calculations on the quenching process of Na(3p) + H_2_ → Na(3s) + H_2_ [23], and the calculated rate constant implied that the quenching efficiency is slightly enhanced when the collision temperature increases. 

Although the NaH_2_ reactive system has received extensive concern in the field of molecular reaction dynamics, previous research mainly focused on the collisions between the electronically excited Na atom and H_2_ or its isotopic variant. However, the microscopic dynamics of the ground-state Na(3s) + H_2_ reaction have not been reported experimentally or theoretically due to the high endothermicity, so understanding this important elementary reaction system is still incomplete. To this end, a global and high-precision ground-state NaH_2_ PES is represented using the NN model based on a large number of high-level ab initio data. Moreover, the state-to-state quantum dynamics calculations of the Na(^2^S) + H_2_(*v*_0_ = 0, *j*_0_ = 0) → NaH + H reaction are carried out using the TDWP method to study the microscopic reaction mechanism in detail. The newly constructed PES can be used to carry out further dynamics studies, such as the effects of vibrational excitations, isotopic substitutions, and spatial alignment of reactant molecules, and the presented quantum dynamics results on the Na(^2^S) + H_2_ reaction could guide the corresponding experimental study at the state-resolved level. 

## 2. Results and Discussion

### 2.1. Characteristics of the Ground-State NaH_2_ PES

**Figure 1:**
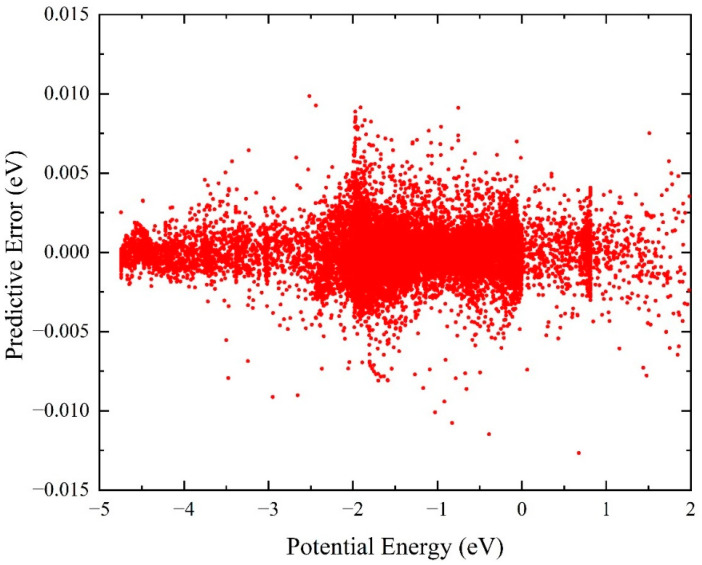
Distribution of the predictive errors of 21,873 ab initio data for the fitting of ground-state NaH_2_ PES.

The distribution of fitting error is a crucial factor in evaluating the accuracy of a reactive PES. To ensure that the constructed PES is suitable for the reaction dynamics calculations, 21,873 ab initio points, including 90% training data and 10% test data, which cover a large coordinate space, were selected to fit the NaH_2_ PES. The distribution of training or test errors obtained by the difference between the fitting values and the original ab initio data of all the points is displayed in Figure 1, and the potential energy value corresponds to the triatomic Na-H-H dissociation limit. It can be seen that the constructed NN keeps a minimal predictive error in the entire potential energy space, especially for energy less than 3.0 eV, which is the crucial dynamically relevant region. The global predictive root-mean-square error (RMSE) is only 1.38 meV, and the maximum absolute predictive error is 12.7 meV. There are only four points with an absolute predictive error larger than 10 meV, and the percentage of points with an absolute error of less than 5 meV can reach 99.1%. Therefore, the constructed ground-state NaH_2_ PES is accurate enough for the dynamics calculations on the Na(^2^S) + H_2_ reactive system. Furthermore, the fitting PES also features strong generalization performance, since the dataset includes 10% of test points. 

**Figure 2:**
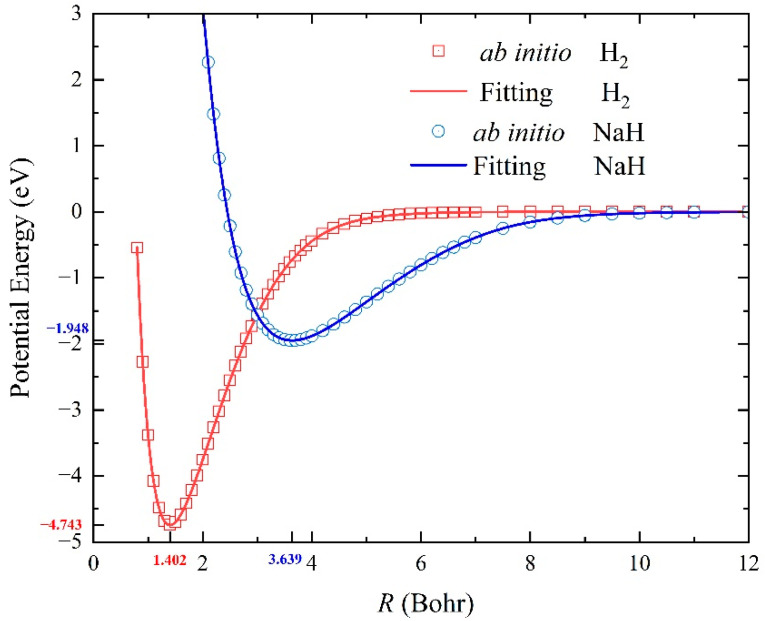
Comparison of the PECs of H_2_(X^1^Σ_g_^+^) and NaH(X^1^Σ^+^) between the results obtained on the ground-state NaH_2_ PES and the original ab initio data.

For a reliable three-body PES, it is required to accurately reproduce the two-body potential energy curve when one atom is placed far away. Table 1 shows the molecular constants of the two diatomic molecules of the ground-state H_2_ and NaH, including the equilibrium distance *R_e_*, dissociation energy *D*_e_, vibrational frequencies *ω_e,_* and anharmonicity coefficients *ω_e_x_e_*. The molecular constants are determined by putting one atom away from the diatomic molecule to sample data points on the three-body NN PES and then fitting the two-body potential energy curves based on these points by the least squares method. The molecular constants of H_2_(X^1^Σ_g_^+^) and NaH(X^1^Σ_g_) molecules determined on the constructed NN PES are consistent with the previous experimental data [25,26], suggesting that the fitting ground-state NaH_2_ PES are accurate enough for describing the two-body potentials and the corresponding rovibrational energy levels of the reactant and product molecules. Figure 2 shows the diatomic potential energy curves (PECs) of H_2_(X^1^Σ_g_^+^) and NaH(X^1^Σ_g_) molecules obtained on the NN PES compared with the ab initio data. It is clear that the two-body PECs of reactant and product molecules are very smooth and are in agreement with the original ab initio points as well, further implying that the fitting PES can accurately reproduce the two-body potentials. 

**Figure 3:**
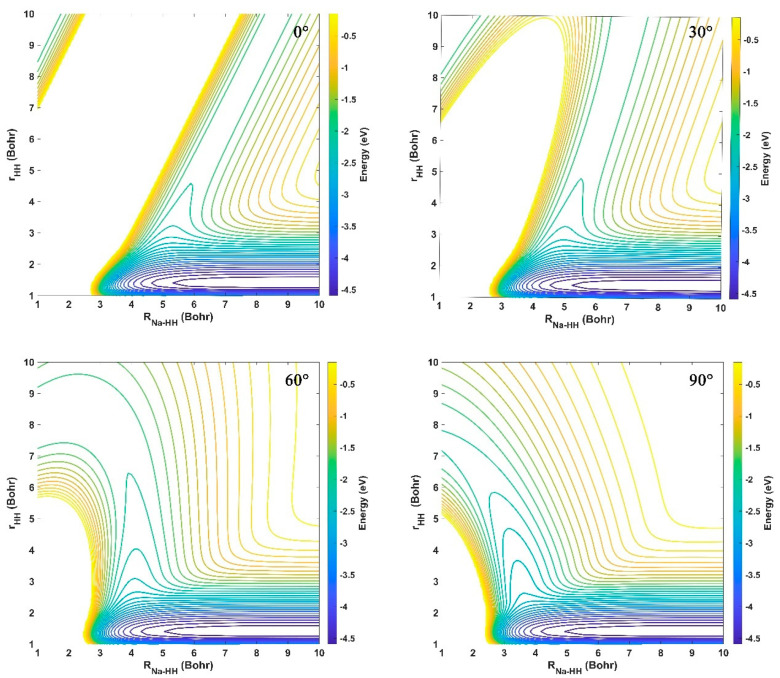
Contours of the fitting ground-state NaH_2_ PES at four insertion angles (*θ* = 0°, 30°, 60°, and 90°) in the reactant Jacobi coordinates.

Figure 3 displays the contour maps of the ground-state NaH_2_ PES in the reactant Jacobi coordinates fixed at four different insertion angles (*θ* = 0°, 30°, 60°, and 90°). *r* and *R* are the HH bond length and the distance between the Na atom and the center of mass of HH, respectively, and *θ* represents the angle between *R* and *r*_._ For each insertion angle, there is a valley with an energy of −4.743 eV at the bottom, which corresponds to the reactant channel. It is clear that the energy of all regions is larger than the one of the H_2_ channel, and no well or barrier is presented, suggesting that the Na atom is subject to repulsive interactions of the H_2_ molecule. The topography of PES features a smooth hump and changes fast when the HH bond is elongated before the formation of the NaH molecule, especially for small insertion angles. When *θ* = 90°, the ground-state NaH_2_ system is at *C_2v_* symmetry, and the repulsive potential reaches the maximum. This is because the ground state 1^2^*A*′ and the first excited state 2^2^*A*′ belong to the same symmetry in the *C_s_* point group, and the two electronic states usually do not cross or accidentally degenerate. However, at *C_2v_* geometry, the lowest two states are changed to ^2^*A*_1_ and ^2^*B*_2_ symmetries, respectively, and avoided crossing arises connecting the two surfaces, around the T-shaped geometry at which there is relatively strong nonadiabatic coupling.

**Figure 4:**
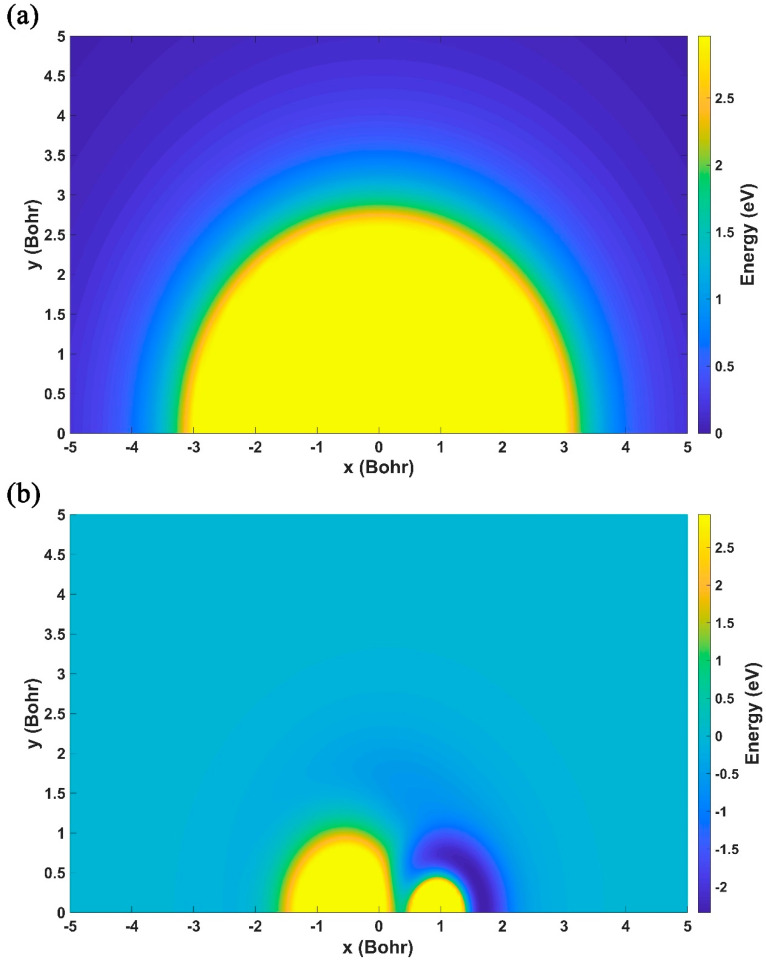
(**a**) Contour map of the fitting ground-state NaH_2_ PES for the Na atom moving around the H_2_ molecule with the bond length fixed at 1.402 Bohr; (**b**) contour map of the fitting ground-state NaH_2_ for the H atom moving around the NaH molecule with the bond length fixed at 3.639 Bohr.

Figure 4a displays the contour map of the constructed ground-state NaH_2_ PES in the case of the Na atom moving around the H_2_ molecule, which is fixed at the equilibrium distance of 1.402 Bohr, and the potential energy is set as zero when the Na atom keeps away from the H_2_ molecule. It can be seen from this contour that the topography of the constructed NN PES is symmetrical relative to *y* = 0, showing excellent exchange symmetry about the two identical H atoms, since the permutation invariant polynomials (PIPs) are selected as the input of NN. The Na atom and H_2_ molecule are always in mutual repulsion, and the repulsive interaction gradually increases as the Na atom approaches the H_2_ molecule, meaning that extra collision energy is required to initiate the title reaction. Figure 4b shows a contour map of one of the H atoms moving around the NaH molecule. The NaH molecule is fixed at its bond length of 3.639 Bohr, and the energy is relative to the dissociation limit of the NaH + H channel. Different from the case of Figure 4a, the H atom is mainly subject to the attractive interactions of the NaH molecule when they become close to each other. There is a well around the H atom and the Na atom, and the potential well around the Na atom is much deeper than the one around the H atom, indicating that another H atom is more easily bounced away from the side of H in the product channel of the Na(3p) + H_2_ reaction.

**Figure 5:**
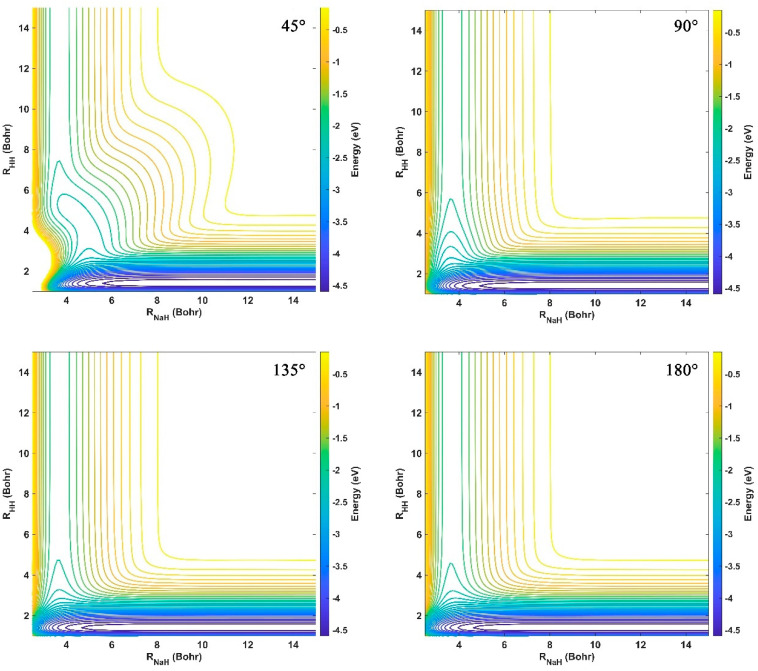
Contours of the fitting ground-state NaH_2_ PES fixed at four approach Na-H-H angles (45°, 90°, 135°, and 180°).

The contour maps of the ground-state NaH_2_ PES at four different approach Na-H-H angles (45°, 90°, 135°, and 180°) are presented in Figure 5. For a PES represented by the NN model, overfitting occurs frequently, especially at long-range regions, which is because the interaction regions feature more complex morphology and structure; whereas, the long-range potential changes slowly, so using the same NN model to fit PES could result in overfitting behaviors in the long-range regions. It can be seen from this set of contours that the constructed NN PES is extremely smooth in the whole coordinate space, and the nonphysical structure is not presented at each fixed angle, meaning that the NaH_2_ PES is globally accurate, and no overfitting occurs during the NN training. There are two valleys on the left and bottom, corresponding to the reactant Na(^2^S) + H_2_ channel and product NaH + H channel, respectively. No well or barrier is presented between the two channels at each approach angle, so the title reaction is dominated by a direct abstraction process. The energy value of the bottom valley is much smaller than that of the left valley, suggesting that the title reaction has a large endothermy. 

**Figure 6:**
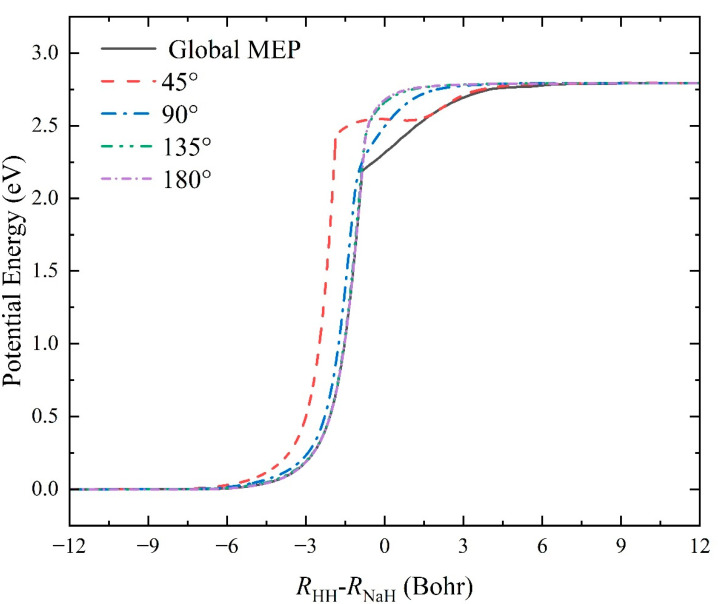
Global MEP and the MEPs of the Na(^2^S) + H_2_(*v*_0_ = 0, *j*_0_ = 0) → NaH + H reaction at four Na-H-H approach angles (45°, 90°, 135°, and 180°) calculated on the fitting ground-state NaH_2_ PES.

To present the properties of the Na(^2^S) + H_2_(*v*_0_ = 0, *j*_0_ = 0) → NaH + H reaction based on the topography of PES more intuitively, the global minimum energy path (MEP) and the MEPs of this reaction at four fixed Na-H-H approach angles (45°, 90°, 135°, and 180°) are calculated and shown in Figure 6. The MEPs are determined by scanning the global ground-state NaH_2_ PES with the step lengths of *ΔR* = 0.01 Bohr and *Δ∠*Na-H-H = 1° at different reactant coordinates of *R*_HH_ − *R*_NaH_ to find the minimum potential energy value. The global MEP features a monotone rising potential energy from the reactant asymptotic region to the product channel, and there is no well or barrier structure, implying that the Na atom directly takes away a H atom of the H_2_ to form the NaH molecule when the collision energy reaches the reaction threshold. At a relatively low collision energy, the Na approaches the H_2_ molecule along the global MEP pathway with the elongation of the HH bond to deprive the H atom, and then, the NaH bond is generated. When the Na-H-H approach angle is fixed at 45°, the MEP features a shallow well with a depth of 0.257 eV at the product region; thus, the reaction process first produces an intermediate in the potential well, and then, the product NaH molecule is formed by the dissociation of the triatomic NaHH complex. For a larger approach angle, the reaction path is similar to the global MEP; namely, it rises smoothly from the Na(^2^S) + H_2_ channel to the NaH + H channel, and the rising slope increases as the Na-H-H angle increases, suggesting that the title reaction prefers to proceed along a relatively small approach angle. In addition, the shape of MEPs at 135° and 180° collision angles is very similar, indicating that the topography of the PES has a small change at the large Na-H-H angle. When the vibrational zero-point energies of H_2_ and NaH molecules are considered, the endothermicity of the title reaction determined by the NN PES is 2.598 eV. The Fortran code of the constructed ground-state NaH2 PES can be founded in the Supplementary Materials.

### 2.2. Quantum Dynamics Calculations

**Figure 7:**
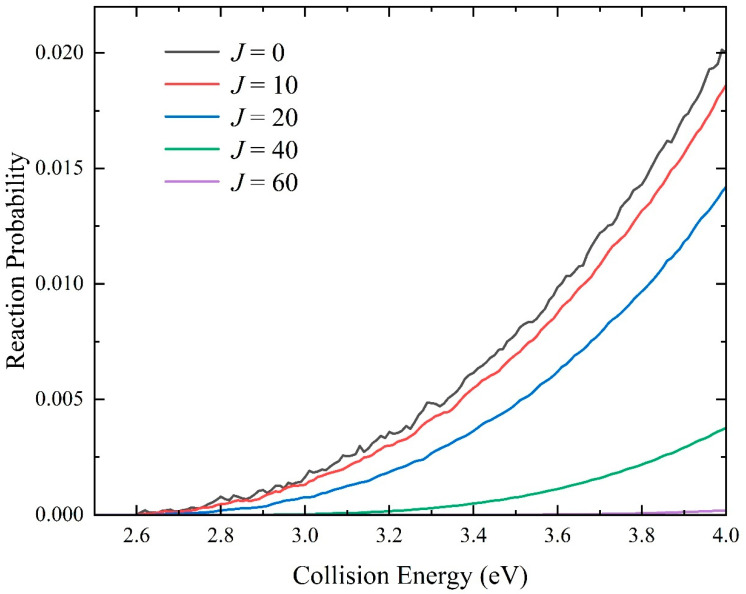
Total reaction probabilities as a function of collision energy for the Na(^2^S) + H_2_(*v*_0_ = 0, *j*_0_ = 0) → NaH + H reaction with five partial waves (*J* = 0, 20, 40, 50, and 60) calculated by the TDWP method on the fitting ground-state NaH_2_ PES.

On this newly constructed ground-state NaH_2_ PES, the quantum dynamics calculations at the state-to-state level of the Na(^2^S) + H_2_(*v*_0_ = 0, *j*_0_ = 0) → NaH + H reaction is implemented using the TDWP method to analyze the microscopic reaction mechanism. Figure 7 shows the collision energy dependence of total reaction probabilities of the title reaction at four different partial waves (*J* = 0, 20, 40, and 60). For the probability curve of *J* = 0, the reaction threshold equals the endothermicity determined by the NN PES, because the global MEP is barrierless. There are tiny oscillations near the threshold, which can be attributed to the generation of a short-lived NaHH complex in the shallow well when the Na atom collides with the H_2_ through a relatively small Na-H-H angle. Additionally, the well could support some bound and quasi-bound states, resulting in the formation of quantum resonance. As the collision energy increases, the amplitude gradually becomes small due to the increasing dominance of the reaction paths with larger collision angles. The reaction threshold increases, and the resonance peaks become wider and, eventually, disappear with the increase in the *J* value, which is because the increasing centrifugal potential barrier hiders the collision process and smooths the effective well. The increasing rate of threshold becomes faster as the total angular momentum quantum number increases due to the directly proportional relationship between the centrifugal potential and *J*(*J* + 1).

**Figure 8:**
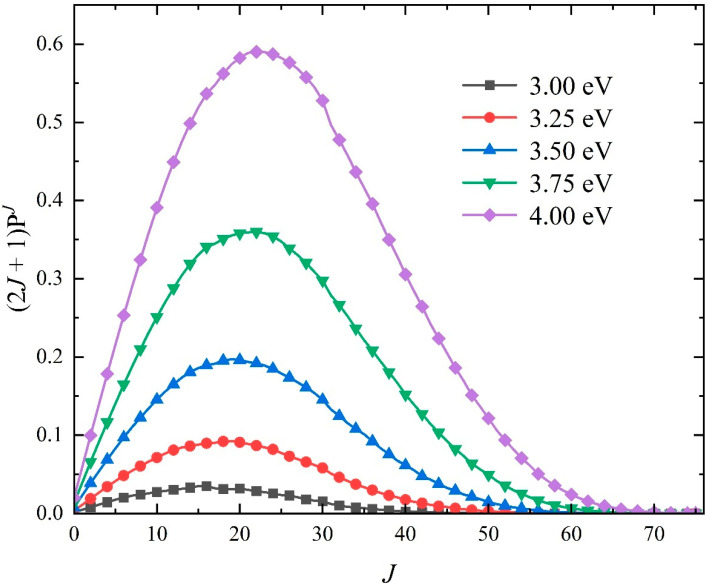
Opacity functions of the Na(^2^S) + H_2_(*v*_0_ = 0, *j*_0_ = 0) → NaH + H reaction at five collision energies (*E_c_* = 3.0, 3.25, 3.5, 3.75, and 4.0 eV) calculated by the TDWP method on the fitting ground-state NaH_2_ PES.

To study the contributions of different partial waves on the total integral cross-sections (ICSs) of the Na(^2^S) + H_2_(*v*_0_ = 0, *j*_0_ = 0) → NaH + H reaction, Figure 8 displays the weighted opacity functions multiplied by (2*J* + 1) at five collision energies (*E_c_* = 3.0, 3.25, 3.5, 3.75, and 4.0 eV). It can be seen that the curves first rise almost linearly with the *J* value and then drop fast near the maximum available partial wave, and the probability distributions feature a single peak and do not present the oscillating, since there is no deep well on the ground-state NaH_2_. Therefore, the title reaction prefers to proceed along a smooth pathway, meaning the nonstatistical dynamics behave for the formation of the product NaH molecule. The distribution peak is located at around *J* = 15–20 in the studied collision energy range, suggesting that the title reaction is dominated by the relatively low partial wave, which corresponds to the small impact parameter in classical reaction dynamics. As the collision energy increases, the peak shifts slightly to the right, implying that the advantage of the reaction pathway with a relatively large impact parameter becomes larger, resulting in the generation of product molecules dominated by a direct approach without a three-body complex forming. 

**Figure 9:**
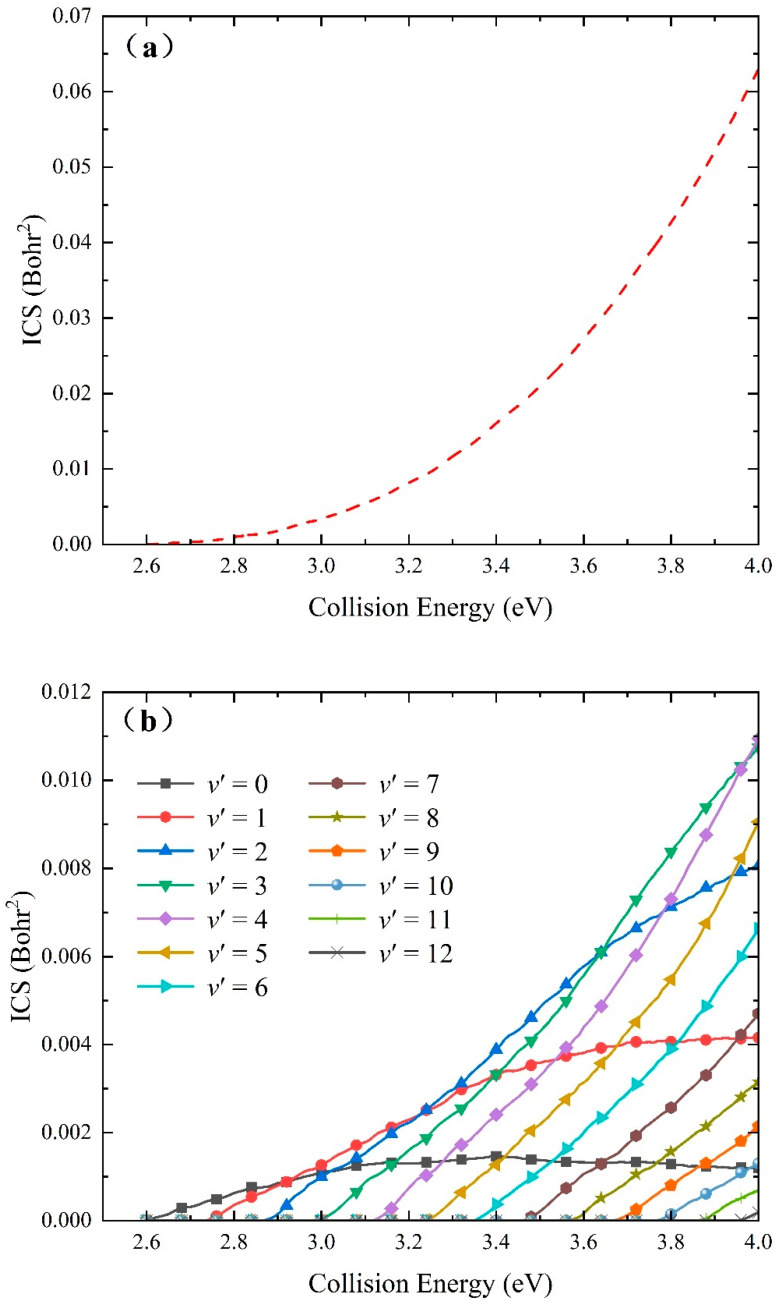
Collision dependence of (**a**) total and (**b**) vibrationally state-resolved ICSs of the Na(^2^S) + H_2_(*v*_0_ = 0, *j*_0_ = 0) → NaH + H reaction calculated by the TDWP method on the fitting ground-state NaH_2_ PES.

The total ICSs of the Na(^2^S) + H_2_(*v*_0_ = 0, *j*_0_ = 0) → NaH + H reaction as a function of collision energy is given in Figure 9a compared with the curves of reaction probability at a certain *J* value; all the oscillating structures are erased by summing over various available partial waves. The total ICSs monotonously increase with the collision energy, conforming to a typical feature of endothermic reactions. The rising slope of total ICSs gradually becomes larger, because the relatively low partial waves have the dominant contribution to the title reaction, causing the total ICS value to rise quickly with the increase in collision energy. To further understand the dynamics mechanisms of the title reaction at the state-to-state level, the collision energy dependence of vibrationally state-resolved ICSs of the product NaH molecule is shown in Figure 9b. In the small collision energy range, the product molecule can be excited to the *v*′ = 12 energy level, revealing the characteristic of a direct reaction. When the collision energy reaches the reaction threshold, only the products of the ground vibrational state are formed, and more available vibrational quantum numbers are sequentially presented as the collision energy increases. There is an obvious vibrational population inversion: the ICS values of highly vibrational excited states (*v*′ = 3–6) are larger than that of the vibrationally ground-state and low-lying excited states when the collision energy exceeds 3.5 eV, suggesting that the proportion of the NaH molecule converted to the vibrational energy from the collision energy becomes larger. It can be seen that the rising slope of the two lowest vibrational states (*v*′ = 0–1) gradually decreases; whereas, the ICSs of high vibrational states (*v*′ = 2–12) increase rapidly with the increase in collision energy. It means that the title reaction along the MEPs with a shallow well mainly forms the low-vibrational NaH molecules, and the product molecules populated at the higher vibrational energy level are generated by the global MEP or a larger Na-H-H collision angle. Therefore, the low-vibrational and high-vibrational NaH molecules are dominated by the short-lived complex-forming mechanism and direct abstraction process, respectively. 

**Figure 10:**
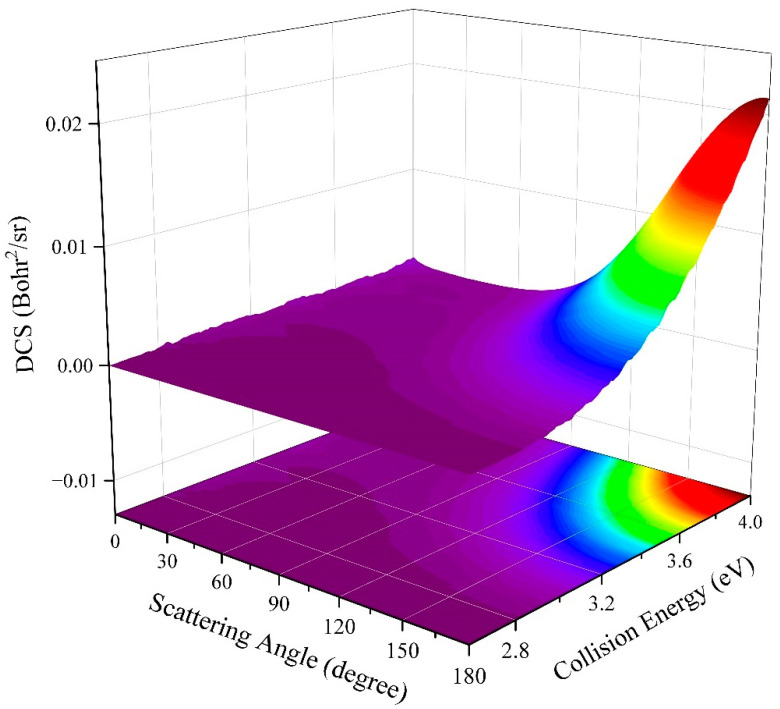
Total DCSs of the Na(^2^S) + H_2_(*v*_0_ = 0, *j*_0_ = 0) → NaH + H reaction as a function of collision energy and scattering angle calculated by the TDWP method on the fitting ground-state NaH_2_ PES.

The differential cross-sections (DCSs) can reveal the microscopic mechanisms of an element reaction more intuitively based on the angle distributions of the product molecules. Figure 10 shows the three-dimensional diagram of the total DCSs of the Na(^2^S) + H_2_(*v*_0_ = 0, *j*_0_ = 0) → NaH + H reaction as a function of the scattering angle and collision energy. At relatively low collision energy, the peaks of the DCSs are located at two polar angles (0° and 180°), and there is a small forward bias, presenting a weak statistical nature, which can be attributed to the contribution of the shallow well on the MEP with a small collision angle. As the collision energy increases, the peak value of forward scattering decreases rapidly and eventually disappears; whereas, the backward scattering becomes more and more obvious, implying that the title reaction is completely dominated by a rebound mechanism when the collision energy exceeds 3.2 eV. This is because the title reaction mainly proceeds along the MEPs with a small Na-H-H angle when the collision energy is slightly larger than the threshold; thus, a short-lived complex is generated, resulting in a nearly forward–backward symmetric angle distribution. As the extra energy increases, more reaction pathways with large rising slopes, as shown in Figure 6, are opened, and the contribution of high partial waves also increases, causing the effective well on the reaction pathways with small collision angles to be smoothed. Therefore, the title reaction includes the short-lived complex-forming mechanism at relatively low collision energy, and a direct H abstraction process plays the dominant role in the whole studied collision energy range. 

## 3. Theory and Method

### 3.1. Ab Initio Calculations

The single-point energy of the ground-state (1^2^A′) NaH_2_ system is calculated at the multireference configuration interaction (MRCI) level [27,28] with the Davidson correction (+Q), and the H atom and Na atom are adopted by AV5Z basis set and WCVQZ basis set [29], respectively. The final molecular orbitals are optimized based on the complete active space self-consistent field (CASSCF) wavefunctions [30,31]. The CASSCF orbitals are calculated with the equally weighted state average for the 1^2^A′, 2^2^A′, and 1^2^A″ electronic state, and 3 valence electrons are included in 6 active orbitals (5*a*′ + 1*a*″). A mass of molecular geometries over a large range within *C_s_* symmetry is selected to ensure the globality of the constructed PES. The configuration space is determined in the Jacobi coordinates. The reactant region is constructed by 0.8 ≤ *r*_HH_/*a*_0_ ≤ 20.0, 0.1 ≤ *R*_Na-HH_/*a*_0_ ≤ 30.0, 0 ≤ *θ* ≤ π/2, and the product region is constructed by 1.8 ≤ *r*_NaH_/*a*_0_ ≤ 20.0, 0.1 ≤ *R*_H-NaH_/*a*_0_ ≤ 30.0, 0 ≤ *θ* ≤ π′. All the ab initio points are calculated using the Molpro 2012 quantum chemistry program [32]. 

### 3.2. PES Fitting

There is increasing interest in structuring molecular PESs using machine learning models [33,34,35,36], and the NN method is the most popular strategy in constructing reactive PESs, because of the unique advantages of high fitting accuracy, strong generalization performance, and fast speed of evaluation of evolution, which has been widely applied to simple reactive, polyatomic, diabatic molecular, and gas−surface scattering systems [37,38,39,40,41,42,43,44,45,46,47,48,49]. Herein, we use the backpropagation NN method to represent the global ground-state NaH_2_ PES. Before the NN fitting, the permutation invariant polynomials (PIPs) [50,51,52] are structured based on the bond length between any two atoms to ensure the fitting PES satisfies the exchange symmetry about the two H atoms. The primary invariants are written as:

(1) $$p_{i}=\exp(\alpha R_{i})$$

where α is an adjustable parameter, and its value is between 0 and 1 to ensure the feature scaling of *p_i_*. Here, α is set as 0.2, which can result in the fitting error decreasing faster by numerous tests. Then, the symmetrized polynomial vector ***S*** = {*S_i_*} is constructed as:

(2) $$S_{1}=(p_{{\mathrm{NaH}}_{1}}+p_{{\mathrm{NaH}}_{2}})/2$$

(3) $$S_{2}=p_{{\mathrm{NaH}}_{1}}\times p_{{\mathrm{NaH}}_{2}}$$

(4) $$S_{3}=p_{\mathrm{HH}}$$

Finally, the vector ***S*** is normalized as:

(5) $$I_{i}=\frac{2(S_{i}-S_{i,\min})}{\left(S_{i,\max}-S_{i,\min}\right)}-1$$

The input of NN selects the vector ***I***, and the corresponding normalized potential energy serves as the output. Two hidden layers with 15 neurons in each layer are selected. Namely, the 3-15-15-1 NN structure is used to fit the global PES, so 315 parameters are needed to optimize the NN model. The transfer function *φ* for the 1–2 and 2–3 layers is the hyperbolic tangent function, and the 3–4 layer is connected by a linear function of identity function, respectively; the analytical form of the fitting PES can be written as:

(6) $$V_{\mathrm{fit}}=\varphi^{\left(3\right)}\left({b_{1}}^{\left(3\right)}+\sum_{i=1}^{15}w_{i1}^{\left(3\right)}\varphi^{\left(2\right)}\left({b_{i}}^{\left(2\right)}+\sum_{j=1}^{15}w_{ji}^{\left(2\right)}\varphi^{\left(1\right)}\left({b_{j}}^{\left(1\right)}+\sum_{k=1}^{3}w_{kj}^{\left(1\right)}I_{k}\right)\right)\right)$$

Here, the RMSE between the ab initio data and predictive results is the cost function to assess the preformation of the training model, and the linking weight *w* and bias *b* between two adjacent neurons with different layers are optimized circularly by the Levenberg–Marquardt algorithm [53].

A total of 21,873 high-level energy points that cover the entire coordinate region are chosen to fit the global ground-state NaH_2_ PES. To avoid overfitting, all the ab initio points are divided into 90% training data and 10% testing data randomly. When the training error decreases slowly or the predictive error starts to increase, the fitting process is required to stop immediately.

### 3.3. TDWP Method

For quantum calculations of simple reactions, the TDWP scheme [54,55,56,57] features strong extensibility and high accuracy, which has been widely used in the quantum dynamics studies of various triatomic systems. Here, we used the TDWP method to calculate the reaction dynamics information of the title reaction based on the newly constructed NN PES. The Hamiltonian of the Na(^2^S) + H_2_ reaction system in the reactant Jacobi coordinates is expressed as:

(7) $$\hat{H}=-\frac{\hbar^{2}}{2\mu_{R}}\frac{\partial^{2}}{\partial R^{2}}-\frac{\hbar^{2}}{2\mu_{r}}\frac{\partial^{2}}{\partial r^{2}}+\frac{{\left(\hat{J}-\hat{j}\right)}^{2}}{2\mu_{R}R^{2}}+\frac{{\hat{j}}^{2}}{2\mu_{r}r^{2}}+\hat{V}(r,R,\theta ),$$

where *j* is the rotational quantum number of the H_2_ molecule, and *J* is the total angle momentum quantum number of the NaH_2_ system. *µ_r_* and *µ_R_* are the reduced masses associated with *R* and *r*, respectively. *V* represents the Na-HH interaction potential that excludes the reference potential of the H_2_. 

In this work, the state-to-state scattering matrix *S^J^* is extracted by the reactant coordinate-based scheme [58,59], and the wave packet is evaluated by the second-order split operator propagator [60]. The state-to-state reaction probability is calculated by the *S^J^*:

(8) $$P_{v\prime j\prime \leftarrow v_{0}j_{0}}^{J}=\frac{1}{2j_{0}+1}{\sum_{K,K_{0}}\left|S_{\nu \prime j\prime K\leftarrow \nu_{0}j_{0}K_{0}}^{J}\right|}^{2},$$

The state-resolved ICSs are calculated by summing over all available partial waves:

(9) $$\sigma_{v\prime j\prime \leftarrow v_{0}j_{0}}=\frac{\pi }{(2j_{0}+1)k_{v_{0}j_{0}}^{2}}\sum_{K}\sum_{K_{0}}{\sum_{J}\left(2J+1\right)\left|S_{\nu \prime j\prime K\leftarrow \nu_{0}j_{0}K_{0}}^{J}\right|}^{2},$$

in which $k_{v_{0}j_{0}}=\sqrt{2\mu_{R}E_{c}}$ is the wave vector in the entrance channel. The state-to-state DCSs can be calculated by:

(10) $$\frac{d\sigma_{\upsilon j\leftarrow \upsilon_{0}j_{0}}(\vartheta ,E)}{d\Omega }=\frac{1}{\left(2j_{0}+1\right)}{\sum_{K}\sum_{K_{0}}\left|\frac{1}{2ik_{\upsilon_{0}j_{0}}}\sum_{J}(2J+1)d_{KK_{0}}^{J}(\vartheta )S_{\nu jK\leftarrow \nu_{0}j_{0}K_{0}}^{J\epsilon }\right|}^{2}$$

where ϑ is the scattering angle, and $d_{KK_{0}}^{J}(\vartheta )$ expresses the reduced rotation matrix element.

In this work, the initial rovibrational state of the reactant H_2_ is set as the rovibrational ground state (*v*_0_ = 0, *j*_0_ = 0). A total of 75 partial waves are calculated for the Na(^2^S) + H_2_(*v*_0_ = 0, *j*_0_ = 0) → NaH + H reaction to ensure that the ICSs and DCSs are convergent when the collision energy is below 4.0 eV. The numerical parameters in the TDWP are determined by numerous convergence tests, as listed in Table 2. 

## 4. Conclusions

In this work, we represent a high-fidelity ground-state NaH PES using the artificial NN method based on 21873 high-level ab initio data. The energy points are calculated by the MRCI + Q method with AV5Z and WCVQZ basis sets for H and Na atoms, respectively, and the global predictive RMSE is only 1.38 meV. The spectrographic constants of H_2_(X^1^Σ_g_^+^) and NaH(X^1^Σ_g_) molecules calculated on the NN PES are consistent with the experimental results. The topography characteristics of the triatomic PES are described in detail, and the global PES is relatively smooth, which does not present obvious well or barrier structures. Based on the newly constructed NN PES, the quantum TDWP calculations on the Na(^2^S) + H_2_(*v*_0_ = 0, *j*_0_ = 0) → NaH + H reaction are performed to analyze the microscopic dynamics mechanism. There are tiny oscillations on reaction probability curves at low partial waves owing to the existence of reaction pathways with a shallow well, and the resonance structures are smoothed for the relatively high *J* value. The total ICSs are mainly contributed by the partial waves of *J* = 15–20 and increase rapidly with the collision energy in the selected collision energy range. The vibrational state-resolved ICSs show an obvious vibrational population inversion, and the corresponding collision energy dependence reveals that the low-vibrational NaH molecules are primarily generated by the dissociation of the triatomic complex; whereas, the products with higher vibrational states are formed by a direct abstraction of the H atom. The total DCS results indicate that the title reaction is dominated by the direct abstraction process, and it includes the short-lived complex-forming mechanism when the collision energy is slightly larger than the reaction threshold. 

## Figures and Tables

**Table 1:** Molecular constants of H_2_(X^1^Σ_g_^+^) and NaH(X^1^Σ^+^).

		NN PES	Experimental Data
H_2_(X^1^Σ_g_^+^)	*R_e_* (Bohr)	1.402	1.401
	*D_e_* (eV)	4.743	4.747
	*ω_e_* (cm^−1^)	4400.1	4401.2
	*ω_e_x_e_* (cm^−1^)	120.3	121.3
NaH(X^1^Σ^+^)	*R_e_* (Bohr)	3.639	3.566
	*D_e_* (eV)	1.948	1.971
	*ω_e_* (cm^−1^)	1170.4	1171.9
	*ω_e_x_e_* (cm^−1^)	19.3	19.7

**Table 2:** Numerical parameters used in the TDWP calculations.

Na(^2^S) + H_2_(*v*_0_ = 0, *j*_0_ = 0) → NaH + H	
Grid ranges and sizes	*R*∈[0.01,25.0], $N_{R}^{\mathrm{tot}}$= 299, $N_{R}^{int}$= 199
	*r*∈[0.1,25.0], $v_{int}$= 249, $v_{asy}$= 11
	$j_{int}$= 109, $j_{asy}$= 19
Initial wave packet	*R*_c_ = 14.0 *∆*_R_ = 0.6 *E*_0_ = 0.5 eV
Propagation time	15,000
Highest *J* value	75

## Data Availability

Dataset available on request from the authors.

## Supplementary Materials

The following supporting information can be downloaded at: https://www.mdpi.com/article/10.3390/molecules29204871/s1, The Fortran code of the constructed ground-state NaH2 PES.
